# Fractal Permeability Model of Newtonian Fluids in Rough Fractured Dual Porous Media

**DOI:** 10.3390/ma15134662

**Published:** 2022-07-02

**Authors:** Shanshan Yang, Mengying Wang, Sheng Zheng, Shuguang Zeng, Ling Gao

**Affiliations:** 1 Three Gorges Mathematical Research Center, China Three Gorges University, Yichang 443002, China; 202107010021019@ctgu.edu.cn (M.W.); zsh@ctgu.edu.cn (S.Z.); ring024@126.com (L.G.); 2 College of Science, China Three Gorges University, Yichang 443002, China; zengshuguang19@163.com

**Keywords:** Newtonian fluid, fractured dual porous media, permeability, rough

## Abstract

Based on the statistical self-similar fractal characteristics of the microstructure of porous media, the total flow rate and permeability of Newtonian fluids in the rough fracture network and rough matrix pores are derived, respectively. According to the connection structure between fractures and pores, the permeability analysis model of fluids in a matrix-embedded fracture network is established. The comparison between the predicted values of the model and the experimental data shows that the predicted values of the permeability of the rough fracture network and the rough matrix pores decrease with the increase in the relative roughness of the fractures and matrix pores, and are lower than the experimental data. Meanwhile, the predicted total flow rate of a rough fractured dual porous media is lower than that of a smooth fractal model and experimental data. In addition, it is also found that the larger the average inclination angle and the relative roughness of the fracture network, the smaller the permeability of the fractured dual porous media, and the relative roughness of the fracture network has a far greater influence on fluid permeability in the fractured dual porous media than the relative roughness of the matrix pores.

## 1. Introduction

Natural porous media, such as oil and gas reservoirs, coal reservoirs, mineral resources, groundwater resources, and rock and soil, not only contain micropores, but also have a large number of large-scale channels and fractures. The study on the seepage characteristics of the fluid in this kind of rough fracture dual porous media has important significance and wide practical application fields, such as the spontaneous self-priming of fluids in rough porous media [1], effective thermal conductivity and heat and mass transfer [2,3], development of tight oil and gas reservoirs, transportation of natural gas in complex pores of tight sandstone [4,5], control of working fluid leakage and reservoir protection in fractured oil and gas reservoirs [6], regular packing in industrial separation processes [7], fractal study on electrolyte diffusion in charged porous media [8], and the improvement of fuel cell performance [9].

Previous studies have shown that the pore size distribution of porous media satisfies the fractal relationship [10,11]. Yu et al. [12] gave the statistical characteristics of porous media based on fractal theory, proposed a unified model suitable for fractal porous media, and gave the criteria for judging whether porous media can be studied by fractal theory. Since the seepage characteristics of fluids in porous media are affected by many factors. Considering the shape, the spatial distribution of pores, and the characteristics of the fluids in rock formations, scholars have proposed various models and methods to simulate fluid flow in the porous medium. Based on fractal features, Yu et al. [13] considered factors such as tortuosity, particle size, pore area, and porosity, and used the box counting method to calculate the fractal dimension, established a bidisperse porous media model and verified its effectiveness. Based on fractal theory, Wang et al. [14] equated fractured rocks with rectangular and elliptical pipes with cross-sections, respectively, and established a prediction model for the total flow rate and effective permeability of power-law fluid in porous media. Zhu et al. [15] used the tree-like fractal bifurcation network to characterize the fracturing fracture morphology of low permeability reservoirs, established the permeability model of the fracturing fracture network with elliptical and rectangular cross-sections, and discussed the influence of fracture bifurcation series, fracture aspect ratio, cross-section shape and other factors on the permeability of the fracture network. The analytical expression of the relative permeability of wet and non-wet phases was derived by Cai et al. [16], based on the fractal characteristics of pore size distribution and tortuosity in porous media. These models have been established based on fractal theory. Zhang et al. [17] combined the multi-scale finite element method with deep learning to simulate the flow of fluids in porous media based on a set of coarse grids and a set of fine grids. Zeng et al. [18] based on capillary bundles and tree-like network models, established apparent permeability models of gas in single capillaries and fractures by considering such factors as dynamic variation of fracture width, water saturation and actual gas effect, and provided the influence of matrix structural parameters on permeability. Although the above studies have made some progress, only one smooth feature is considered.

The fracture network is embedded into the matrix porous medium to form the so-called dual porous media, and dual porous media is widely present in nature. When exploiting natural gas resources or solving surface pollution problems, dual porosity and dual porous media are involved. Therefore, the transport characteristics of dual-porosity, dual porous media has aroused extensive and lasting research interest. Miao et al. [19] extended the fractal porous media theory to fracture networks and deduced the permeability research model of fracture-pore porous media and found that the maximum pore diameter and maximum trace length have a great influence on fluid seepage characteristics. Miao et al. [20] also considered the transfer flow behavior of fluids from matrix pores to fractured media, and proposed a dimensionless permeability model for porous media, finding that the ratio of fracture pressure difference to matrix pore pressure difference has an important impact on fluid flow from matrix pores to fractured media. Based on the topology model and complex network theory, Zhu et al. [21] proposed the power-law distribution of edges with node degree and established the permeability analytical formula of the dual porous medium, and found that the self-similarity index and the clustering coefficient of pore nodes play a decisive role in the permeability characteristics of the dual porous medium. By considering the channeling between the capillary bundle and the fracture, Wu et al. [22] established the effective permeability model of a power-law fluid in a fracture-pore type medium and found that the effective permeability of power-law fluid in a dual porous medium would be affected by the characteristics of the power-law fluid, structural parameters of fractures and pores, and pressure difference. Xiong et al. [23] represented rock fractures and pores as elliptical cross-section microtubules, calculated the permeability of a three-dimensional fracture network under the action of periodic pressure, and analyzed the variation rule of permeability. These are all studies of smooth porous media.

Since smooth surfaces are almost non-existent in nature, any surface (such as skin, desktop, road, glass, and tiny devices), regardless of its smoothness, is microscopically displayed as rough because of a large number of structural defects or geometric defects. Rough topography has an important influence on other transport mechanisms such as the flow resistance of fluids along rough surfaces, so it is necessary to characterize and describe the characteristics of rough surfaces accurately and simply. Qu [24] summarized the description methods of rough fracture structures, including raised height characterization method, joint roughness coefficient method, and fractal dimension method, and got the rough fracture surface characteristic description parameters such as tortuosity, roughness, and inclination. In practical research, fractal dimension, tortuosity, roughness, and inclination are generally used to characterize the rough surface of the rock stratum. Yang et al. [25] simplified the rough element on the surface of the microchannel into a cone and proposed the permeability fractal model of the rough capillary bundle, the effect of relative roughness on microchannel permeability was studied. Xiao et al. [26] derived the fractal model of fluid flow through a single rough curved capillary in fibrous porous media, elucidating the physical properties of fluid flow in a rough fiber porous medium. Liang et al. [27] calculated the immersion depth of the fluid in the curved capillary and used fractal theory to discuss the effect of relative roughness with immersion depth. He et al. [28] introduced tortuosity based on a fractal binary tree model, established a fractal network model of tortuosity fractal binary tree, and obtained the fluidity change law of gas in the shale reservoir fracture network. All the above models studied the permeability of the fluid in a single rough fracture but did not study in-depth the effect of roughness on fluid seepage in dual porous media.

Zhang et al. [29] wrote a calculation program based on the Boltzmann method to simulate the fluid flow in a single fracture with different JRC values and openings, and compared the simulated values with the results of the tortudic modified cubic law without considering roughness, and found that the tortudic modified cubic law predicted the flow with a certain deviation. Wang et al. [30] made 30 fractured rock samples with 10-level roughness (JRC value) and 3 different gap widths made by 3D printing technology into interpenetrating and filled fractured rock samples, and tested their permeability. Experiment data show that when the confining pressure is small, the greater the roughness, the greater the difference in fracture permeability of rock samples with different gap widths. Based on ten Barton standard lines, Li et al. [31] used COMSOL numerical simulation software to establish a rough single-fracture seepage model to detect the change of seepage velocity under different roughness. The results showed that the larger the roughness coefficient (JRC), the smaller the maximum and average flow velocity of fluid seepage. They described the effect of roughness on fluid seepage qualitatively, and considered that roughness is one of the important factors to be considered in the study of fluid seepage characteristics in fractures. According to the actual physical significance, roughness can reduce the actual fluid flow space, increase the flow resistance, and then reduce the permeability. Therefore, roughness is a factor that cannot be ignored.

Considering the roughness factor can result in a more comprehensive analysis of the permeability variation trend of porous media, and can also improve the accuracy of the model. At the same time, the roughness model can also be extended to other research fields, such as the development of shale gas, the study of thermal conductivity of porous media, and the development of tight oil and gas reservoirs. The literature [29,30,31] did not quantitatively describe the roughness. In order to quantitatively study the influence of roughness on permeability variation trend, based on the fractal theory and the basic formula of fluid mechanics, the permeability model of rough dual porous media is established. In Section 2, the fractal theory of fracture and pore size distribution in fractured dual porous media is presented. In Section 3, the rough surface of the fractured dual-porous media is characterized, and the specific expression of relative roughness is given. In Section 4, the permeability model of coarse fractured dual porous media is derived. In Section 5, the effectiveness of the model is verified by comparing the predicted values with experimental data, and the influence of structural parameters on the permeability of fractured porous media is studied. In Section 6, the conclusions of this study are given.

## 2. Fractal Theory of Fractured Dual Porous Medium

Many studies have shown that the pore size distribution of porous media meets the fractal scale law [10,11,12,32,33]. Yu et al. [33] gave the fractal scale relation of pore size distribution. Miao et al. [19] extend the fractal scale rate into the fracture network and give the fractal scale relationship of the distribution of fracture trace length. In this section, the fractal scale law is further extended to the fractured dual porous media, and the fractal relations satisfied by pore diameter and fracture trace length are given, respectively.

**Figure 1:**
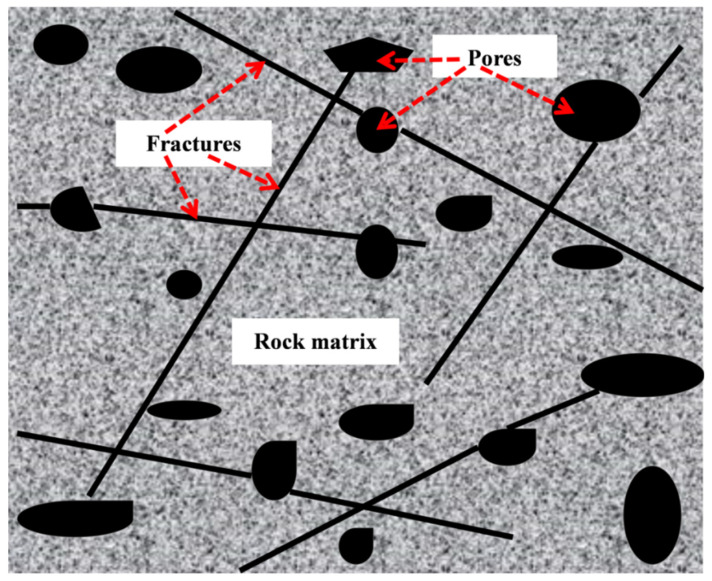
The cross-section structure diagram of the representative elementary volume of the fracture network.

A fractured double porous medium is composed of a fracture network and porous matrix, because the representative elementary volume of the fracture network is much larger than that of the matrix pore [15,19], so the following research is based on the representative elementary volume of the fracture network. Figure 1 is the cross-section structure diagram of the representative elementary volume of the fracture network.

**Figure 2:**
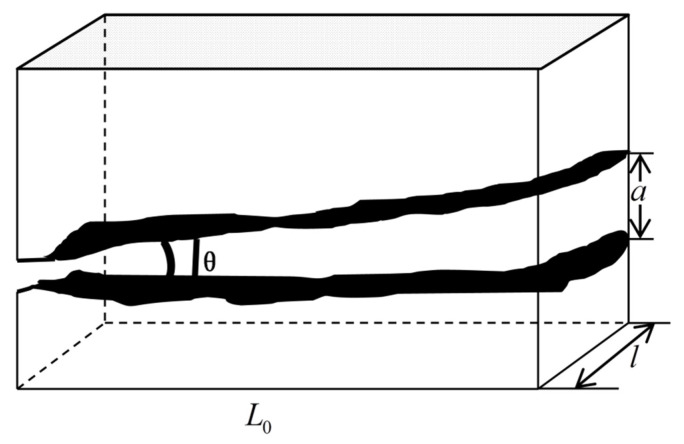
Model characterization of a single fracture.

**Figure 3:**
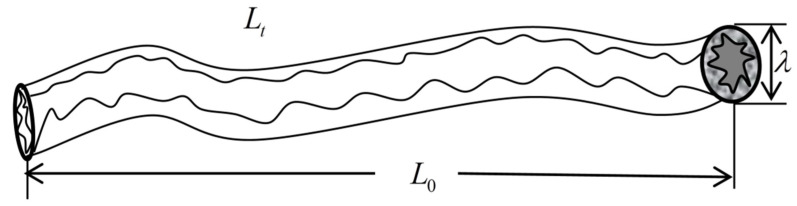
Model characterization of a single capillary bundle.

Due to the difference in geological structure, the diagenetic environment, and other factors, the fractures and matrix pores have different morphological characteristics, and their cross-sections may be: rectangular, polygonal, circular, ellipse, etc. For fractured dual porous media, to simplify the model, it is assumed that the cross-section of the fracture is rectangular and the shape of the fracture is represented by a cuboid. The cross-section of the matrix pores is circular, and its structure is characterized by a capillary bundle model. Figure 2 is the characterization diagram of the rough fracture, the fracture opening is a, the fracture trace length is l, and the fracture inclination angle is θ. Figure 3 shows the characterization diagram of the rough capillary, the diameter of the capillary is  λ, the characteristic length is $L_{0}$, and the actual length is $L_{t}$. Additionally, the fractal theory of fractures and pores is established based on Figure 2 and Figure 3, respectively.

### 2.1. Fractal Theory of Fracture Networks

Since the trace length of fractures satisfies the fractal scaling law [12,19], its power law expression can be written as [33]:

(1) $$N\left(L\geq l\right)\propto l^{-D_{f}}\;,\;l_{\min}<l<l_{\max}$$

where N(l) represents the total number of fractures whose trace lengths are greater than or equal to l, and $l_{\min}$ and $l_{\max}$ represent the minimum and maximum lengths of fracture traces in the fracture network, respectively. $D_{f}$ represents the fractal dimension of fracture trace length in two dimensions, $0<D_{f}<2$, in three dimensions, $0<D_{f}<3\;$. According to the fractal scaling relationship [13,19], Equation (1) can be further rewritten as:

(2) $$N\left(L\geq l\right)=kl^{-D_{f}}$$

where k is the scale factor and an unknown quantity, $l_{\min}\leq l\leq l_{\max}$. Due to the large number of fractures in the fracture network, Equation (2) can be approximated as a continuously differentiable equation, then the number of fractures in [l,l+dl] is:

(3) $$-dN\left(l\right)=kD_{f}l^{-\left(D_{f}+1\right)}dl$$

where the negative sign indicates that the number of fractures decreases with the increase in fracture trace length, which is the same as the actual situation. The total number of fracture trace lengths between $l_{\min}$ and $l_{\max}$ is denoted as $N_{t}$. According to Equation (3), there holds:

(4) $$\frac{-dN\left(l\right)}{N_{t}}=\frac{k}{N_{t}}D_{f}l^{-\left(D_{f}+1\right)}dl=f\left(l\right)dl$$

where f(l) is the trace length of the fracture, that is:

(5) $$f\left(l\right)=\frac{k}{N_{t}}D_{f}l^{-\left(D_{f}+1\right)}$$

normalized by the probability density function, there holds:

(6) $$\int_{l_{\min}}^{l_{\max}}f\left(l\right)dl=\frac{k}{N_{t}}l_{\min}{}^{-D_{f}}\left[1-{\left(\frac{\;l_{\min}}{l_{\max}}\right)}^{D_{f}}\right]\;=1$$

and in porous media there is $l_{\min}/l_{\max}<10^{-2}$, i.e., $l_{\min}<<l_{\max}$, there holds:

(7) $$\frac{k}{N_{t}}l_{\min}{}^{-D_{f}}\;=1$$

the expression of k is:

(8) $$k=N_{t}l_{\min}{}^{D_{f}}$$

substituting Equation (8) into Equation (5), the exact expression for the probability density function is:

(9) $$f\left(l\right)=D_{f}l_{\min}{}^{D_{f}}l^{-\left(D_{f}+1\right)}$$

Yu et al. [13,34] gave the total number of pores when the size distribution of pore diameters satisfies the fractal scaling rate, Majumdar et al. [35] gave the total number of rough spots on the engineering surface, When studying the pores of porous media, it can be compared with the contact points on engineering surfaces, so the total number of fractures is:

(10) $$N_{t}=\left(\frac{l_{\max}}{l_{\min}}\right){\;}^{D_{f}}\;$$

substituting Equation (10) into Equation (8), we obtain the exact value of the proportionality coefficient k:

(11) $$k=l_{\max}{}^{D_{f}}$$

then substitute Equation (11) into Equation (3) to obtain the exact fractal scaling rate satisfied by the fracture trace length:

(12) $$-dN\left(l\right)=D_{f}l_{\max}{}^{D_{f}}l^{-\left(D_{f}+1\right)}dl$$

Yu et al. [12,34] presented the relationship between porosity and fractal dimension when studying fractal scaling rate of porous media. Then, the fractal dimension of the fracture trace length can be defined as:

(13) $$D_{f}=d_{E}-\frac{\ln\phi_{f}}{\ln\left(l_{\min}/l_{\max}\right)}$$

where $d_{E}$ is Euclid dimension, $d_{E}$ is 2 in two dimensions, $d_{E}$ is 3 in three dimensions, $\phi_{f}$ represents the porosity of the fracture network, and refers to the ratio of the total area $A_{pf}$ of fracture pores to the total area $A_{f}$ of cross-section in the cross-section of the fracture network representative elementary volume. According to Equation (13), the expression of porosity $\phi_{f}$ of the fracture network can be written as:

(14) $$\phi_{f}={\left(l_{\min}/l_{\max}\right)}^{d_{E}-D_{f}}$$

at the same time, according to the physical meaning of fracture network porosity $\phi_{f}$, $\phi_{f}$ can also be defined as [36]:

(15) $$\phi_{f}=A_{pf}/A_{f}$$

where $A_{f}$ is the cross-sectional area of the representative elementary volume, $A_{pf}$ represents the total area of fractured pores in that area. According to Equation (12), the total area $A_{pf}$ of fracture pores on the cross-section of the representative elementary volume can be calculated as [36]:

(16) $$A_{pf}=-\int_{l_{\min}}^{l_{\max}}a\cdot ldN=\frac{\beta D_{f}l_{\max}^{2}}{2-D_{f}}\left(1-\phi_{f}\right)$$

then substitute Equation (16) into Equation (15), and the cross-sectional area $A_{f}$ of the fracture network representative elementary volume is obtained as [36]:

(17) $$A_{f}=\frac{A_{pf}}{\phi_{f}}=\frac{\beta D_{f}l_{\max}^{2}}{2-D_{f}}\frac{\left(1-\phi_{f}\right)}{\phi_{f}}$$

where β=a/l, l is the length of the fracture, a is the opening of the fracture. According to Equation (12), the total trace length of the fractures in the feature element body is:

(18) $$\begin{array}{ll} L_{total} & =-\int_{l_{\min}}^{l_{\max}}ldN\left(l\right)=\int_{l_{\min}}^{l_{\max}}D_{f}l_{\max}^{D_{f}}l^{-D_{f}}dl\\ & =\frac{D_{f}}{1-D_{f}}l_{\max}\left[1-{\left(\frac{l_{\min}}{l_{\max}}\right)}^{1-D_{f}}\right] \end{array}$$

according to Equation (14), Equation (18) can be further simplified as:

(19) $$L_{total}=\frac{D_{f}}{1-D_{f}}l_{\max}\left(1-\phi_{f}{}^{\frac{1-D_{f}}{2-D_{f}}}\right)$$

In a two-dimensional fracture network, the fracture areal density D is defined as [37]:

(20) $$D=\frac{L_{total}}{A_{f}}$$

substituting Equations (17) and (19) into Equation (20), the expression of fracture areal density D is [36]:

(21) $$D=\frac{\left(2-D_{f}\right)\left(1-\phi_{f}{}^{\frac{1-D_{f}}{2-D_{f}}}\right)\phi_{f}}{\beta l_{\max}\left(1-D_{f}\right)\left(1-\phi_{f}\right)}$$

Equation (21) shows that the areal density of the fracture network is a function of the maximum length $\;l_{\max}$ of the fracture, and the proportional coefficient β, the fracture porosity $\phi_{f}$, and the fractal dimension $D_{f}$ of the fracture length.

### 2.2. Fractal Theory of Matrix Pores

Considering the matrix porous medium as a curved capillary whose pore diameter satisfies the fractal scaling law [13,34], the total number N(λ) of pores with diameters greater than or equal to λ meets the fractal scale rate:

(22) $$N\left(\lambda^{\prime }\geq \lambda \right)={\left(\lambda_{\max}/\lambda \right)}^{D_{p}}\;,\;\;\lambda_{\min}<\lambda <\lambda_{\max}$$

where $\lambda_{\min}$ and $\lambda_{\max}$ are the minimum and maximum values of pore diameter, respectively. $D_{p}$ is the fractal dimension of pore diameter, and its value range is the same as that of fracture length. The total number of pores is:

(23) $$N_{t}\left(\lambda \geq \lambda_{\min}\right)={\left(\lambda_{\max}/\lambda_{\min}\right)}^{D_{p}}$$

due to the large number of matrix pores, Equation (23) can also be regarded as continuously differentiable functions. The number of pores in [λ,λ+dλ] is:

(24) $$-dN\left(\lambda \right)=D_{p}\lambda_{\max}^{D_{p}}\lambda^{-\left(D_{p}+1\right)}d\lambda$$

where the negative sign indicates that the total number of pores decreases with the increase in pore diameter, which is the same as the actual situation. According to Equations (23) and (24), the probability density function of pore diameter is:

(25) $$f\left(\lambda \right)=D_{p}\lambda_{\min}^{D_{p}}\lambda^{-\left(D_{p}+1\right)},\;$$

According to the relationship between porosity and fractal dimension [12,34], the fractal dimension $D_{p}$ of the pore diameter can be defined as:

(26) $$D_{p}=d_{E}-\frac{\ln\phi_{m}}{\ln\left(\lambda_{\min}/\lambda_{\max}\right)}$$

where $d_{E}$ is Euclidean dimension, $\phi_{m}$ is the porosity of the matrix pores, which refers to the ratio of the total pores area $A_{p}$ to the cross-sectional area $A_{m}$ in the cross-section of matrix representative elementary volume, according to Equation (26). The effective porosity of matrix porous media can be expressed as [12,34]:

(27) $$\phi_{m}={\left(\frac{\;\lambda_{\min}}{\lambda_{\max}}\right)}^{d_{E}-D_{p}}$$

at the same time, according to the physical meaning of matrix porosity $\phi_{m}$, $\phi_{m}$ can be expressed as [36]:

(28) $$\phi_{m}=\frac{A_{p}}{A_{m}}$$

According to Equation (24), the total pore area on the cross-section of matrix representative elementary volume can be calculated as:

(29) $$A_{p}=-\int_{\lambda_{\min}}^{\lambda_{\max}}\frac{\pi \lambda^{2}}{4}dN=\frac{\pi D\lambda_{\max}^{2}\left(1-\phi_{s}\right)}{4\left(2-D_{p}\right)}$$

according to Equation (28), the cross-sectional area of matrix representative elementary volume is expressed as:

(30) $$A_{m}=\frac{A_{p}}{\phi_{m}}=\frac{\pi D_{p}\lambda_{\max}^{2}}{4\left(2-D_{p}\right)}\frac{1-\phi_{m}}{\phi_{m}}$$

The average diameter of pores according to Equations (24) and (25) is:

(31) $$\bar{\lambda }=\int_{\lambda_{\min}}^{\lambda_{\max}}\lambda f\left(\lambda \right)d\lambda =\frac{D_{p}}{\left(D_{p}-1\right)}\lambda_{\min}\left[1-{\left(\frac{\lambda_{\min}}{\lambda_{\max}}\right)}^{D_{p}-1}\right]$$

The actual length $L_{t}\left(\lambda \right)$ and characteristic length $L_{0}$ of the capillaries satisfy the relation [12,34]:

(32) $$L_{t}\left(\lambda \right)=\lambda^{1-D_{T}}L_{0}^{D_{T}}$$

The tortuosity is defined as [38]:

(33) $$\tau =\frac{L_{t}}{L_{0}}={\left(\frac{L_{0}}{\lambda }\right)}^{D_{T}-1}$$

The average tortuosity is defined as [39]:

(34) $$\bar{\tau }=\frac{1}{2}\left[1+\frac{1}{2}\sqrt{1-\phi_{m}}+\sqrt{1-\phi_{m}}\frac{\sqrt{{\left(\frac{1}{\sqrt{1-\phi_{m}}}-1\right)}^{2}+\frac{1}{4}}}{1-\sqrt{1-\phi_{m}}}\right]$$

The tortuosity fractal dimension $D_{T}$ can be defined as [34,40]:

(35) $$D_{T}=1+\frac{\ln\bar{\tau }}{\ln\frac{L_{0}}{\bar{\lambda }}}$$

where $\bar{\tau }$ is the average tortuosity of the capillary, and $L_{0}$ is the characteristic length of the capillary. $\bar{\lambda }$ is the average diameter of the pores, $1<D_{T}<2$ in two-dimensional space, $1<D_{T}<3$ in three-dimensional space. When $D_{T}=1$, the capillary is straight, the greater the $D_{T}$, the greater the bending degree of the capillary.

## 3. Characterization of Rough Surfaces of Fractured Double Porous Media

Qu et al. [24] presented the description methods of rough surface: fractal dimension method, convex height characterization method, tortuosity, roughness and inclination angle. Yang et al. [25] used the convex height method to represent rough elements with cones. He et al. [28] use tortuosity to characterize rough surfaces, They both quantitatively studied the effect of rough surfaces on permeability. In this section, the cone-shaped roughness element model is introduced into the fractured dual porous media, and the roughness representation of fracture and pore surface is given, respectively, according to the distribution of roughness element on the fractured rock surface meets the fractal scale rate [41,42].

### 3.1. Characterization of Rough Surfaces of Fracture Networks

In the following research, Rough elements of rough fracture surfaces are approximated as cones [25,43,44], analogous to Equations (1) and (22), the distribution of the bottom diameter d of the cone follows the fractal scaling law [41,42]:

(36) $$N\left(d^{\prime }\geq d\right)={\left(d_{\max}/d\right)}^{D_{s}}$$

Equation (36) is the fractal scaling law satisfied by the cumulative number N(d) of a rough element whose bottom diameter is greater than or equal to d. Due to a large number of rough elements, Equation (36) can be considered continuous and differentiable. Differential with Equation (36), the number of rough element bottom surface diameter in [d,d+dd] is:

(37) −dN(d)=DdsmaxDsd−(Ds+1)dd

where $D_{s}$ is the fractal dimension of the bottom diameter of the cone, and the larger $D_{s}$, the larger the bottom diameter of the rough element, in two dimensions, $1<D_{s}<2$, in three dimensions, $1<D_{s}<3$. By analogy with Equations (13) and (26), $D_{s}$ can be defined as [12,34]:

(38) $$D_{s}=d_{E}-\frac{\ln\phi_{s}}{\ln\left(d_{\min}/d_{\max}\right)}$$

where $\phi_{s}$ denotes the percentage of the total bottom area $S_{1}$ of all cones to the total surface area $S_{0}$ of fractures in a fracture network representative elementary volume, and $d_{E}$ is the Euclidean dimension. According to Equation (38), $\phi_{s}$ is [12,34]:

(39) $$\phi_{s}={\left(d_{\min}/d_{\max}\right)}^{d_{E}-D_{s}}$$

At the same time, according to the physical meaning of $\phi_{s}$, $\phi_{s}$ can also be defined as [25]:

(40) $$\phi_{s}=\frac{S_{1}}{S_{0}}$$

where $S_{1}$ is the total undersurface area of all cones in the representative elementary volume of the fracture network, and $S_{0}$ is the total area of the fracture surface. The ratio of the height of the cone to the diameter of the base is denoted as σ, that is:

(41) σ=h/d

where d is the bottom diameter of the cone, and h is the height of the cone. The bottom area $S_{i}$ and volume $V_{i}$ of a small cone can be expressed as:

(42) $$S_{i}=\frac{\pi d_{i}{}^{2}}{4}$$

(43) $$V_{i}=\frac{1}{3}S_{i}h_{i}=\frac{\pi d_{i}^{3}\sigma }{12}$$

According to Equation (37), the total volume $V_{t}$ and the total bottom area $S_{1}$ of all cones in the fracture network representative elementary volume are:

(44) $$V_{t}=-\int_{d_{\min}}^{d_{\max}}V_{i}dN=\frac{\pi \sigma }{12}\frac{D_{s}}{3-D_{s}}d_{\max}^{3}\left(1-{\left(\frac{d_{\min}}{d_{\max}}\right)}^{3-D_{s}}\right)$$

(45) $$S_{1}=-\int_{d_{\min}}^{d_{\max}}S_{i}dN=\frac{\pi }{4}\frac{D_{s}d_{\max}^{2}}{2-d}\left(1-\phi_{s}\right)$$

According to Equations (40) and (45), the total area of the fracture surface is:


(46) $$S_{0}=\frac{S_{1}}{\phi_{s}}=\frac{\pi }{4\phi_{s}}\frac{D_{s}d_{\max}^{2}}{2-d}\left(1-\phi_{s}\right)$$

According to the total volume expression by Equation (44) for all cones and the total area expression by Equation (36) for the fracture surface, the effective average height of the cone can be expressed as [25,43,44,45]:

(47) $${\bar{h}}_{s}=\frac{V_{t}}{S_{0}}=\frac{\sigma \phi_{s}d_{\max}}{3}\frac{2-D_{s}}{3-D_{s}}\frac{1-{\left(d_{\min}/d_{\max}\right)}^{3-D_{s}}}{1-\phi_{s}}$$

Then, the relative roughness of the fracture surface is:

(48) $$\varepsilon_{r}=\frac{2{\bar{h}}_{s}}{a}=\frac{2\sigma \phi_{s}d_{\max}}{3a}\frac{2-D_{s}}{3-D_{s}}\frac{1-{\left(d_{\min}/d_{\max}\right)}^{3-D_{s}}}{1-\phi_{s}}$$

In Equation (48) are functions of fracture opening a, fractal dimension $D_{s}$ of rough element bottom diameter, maximum value $d_{\max}$ and minimum value $d_{\min}$ of rough element bottom diameter, and proportional coefficient σ. When the effective average height of the small cone h=0, i.e., σ=0, the fracture surface is smooth, i.e., $\varepsilon_{r}=0$. This corresponds to the actual physical meaning.

### 3.2. Characterization of Rough Surfaces of Matrix Pores

Similar to the characterization of the rough elements of the fracture network, the surface rough masses of the curved capillary channel can also be approximated as cones [25,43,44], according to Equations (44) and (46). The effective average height of rough elements on rough capillaries can be expressed as:

(49) $${\bar{h}}_{c}=\frac{V_{t}}{S_{0}}=\frac{\sigma \phi_{s}d_{\max}}{3}\frac{2-D_{s}}{3-D_{s}}\frac{1-{\left(d_{\min}/d_{\max}\right)}^{3-D_{s}}}{1-\phi_{s}}$$

The relative roughness of the rough capillary bundle microchannel is [25,43,44]:

(50) $$\varepsilon_{c}=\frac{{\bar{h}}_{c}}{\lambda /2}=\frac{2\sigma \phi_{s}d_{\max}}{3\lambda }\frac{2-D_{s}}{3-D_{s}}\frac{1-{\left(d_{\min}/d_{\max}\right)}^{3-D_{s}}}{1-\phi_{s}}$$

Equation (50) shows that the greater the effective average height of the rough element, the greater the relative roughness of the capillary channel. When the effective average height of the rough element is zero, the capillary channel surface is smooth.

## 4. Fractal Model of Seepage in Rough Fractured Dual Porous Media

**Figure 4:**
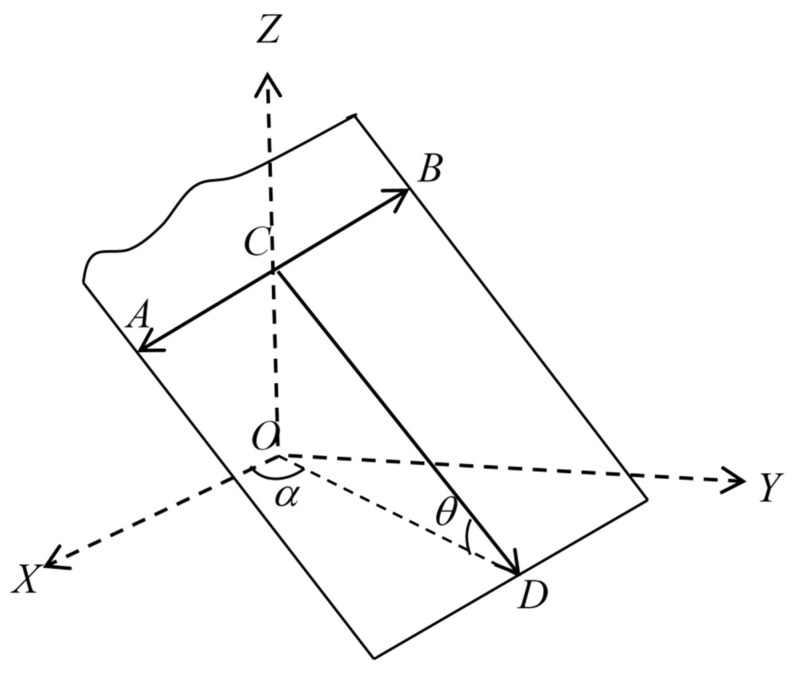
Spatial state of fractures.

Miao et al. [19] established a fracture network permeability model considering the spatial orientation of fractures. The distribution of fractures in space is generally characterized by azimuth, dip angle, and strike [19,46]. The spatial distribution of fractures is shown in Figure 4 below [47], where AB is the fracture strike,  CD is the maximum inclined direction, θ is the fracture inclination, and α is the fracture azimuth. Due to a large number of fractures in complex fracture networks and random distribution patterns in space, to simplify the study of fluid permeability in fractures, in this study, the average inclination angle and average azimuth angle of fractures are generally taken to represent the spatial orientation of the fracture network. It is assumed that the average inclination angle of fractures is θ and the average azimuth angle is α.

Yang et al. [25] proposed a permeability model in a single horizontal rough pore by assuming that the fluid moves along the same direction. In this section, relative roughness, spatial orientation and tortuosity are introduced into the fractured dual porous media to obtain the seepage theory of fractured dual porous media.

### 4.1. Seepage Characteristics of Rough Fracture Networks

**Figure 5:**
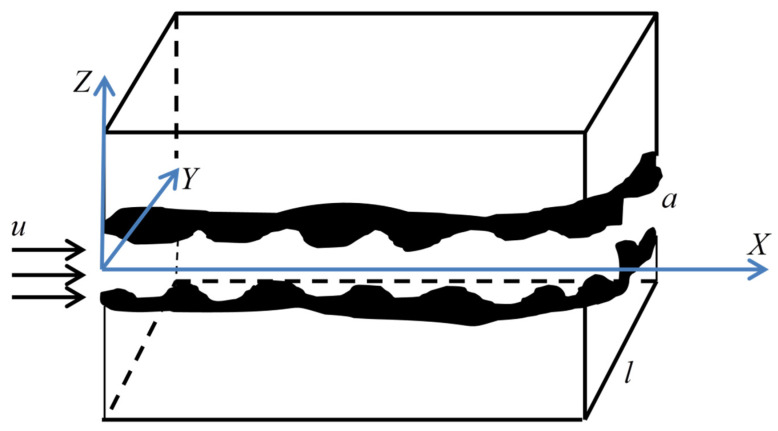
Spatial coordinates of horizontal rough fractures.

Firstly, the horizontal rough single fracture is studied. It is assumed that the fluid flows in the same direction, namely the X direction, and only the velocity component in the X direction is not zero, and the pressure gradient along the X direction remains unchanged. The spatial Cartesian coordinate system shown in Figure 5 is established for research [25]. The fracture is approximately regarded as a cuboid, and the width of the cuboid is the trace length l, the fracture opening is a.

According to the Hagen–Poiseuille law, the fluid motion equation in fracture microchannel is [48]:

(51) $$\frac{\partial^{2}u}{\partial z^{2}}+\frac{\partial^{2}u}{\partial y^{2}}=\frac{1}{\mu }\frac{dp}{dx}$$

where dp/dx is the pressure gradient along the flow direction X, u is the flow velocity along the X direction, and μ is the viscosity coefficient. Since the fracture opening is much smaller than the fracture trace length, namely a≪l, Equation (51) is simplified to:

(52) $$\frac{d^{2}u}{dz^{2}}=\frac{1}{\mu }\frac{dp}{dx}$$

Assuming that the fracture microchannel is symmetric, the non-slip boundary condition is [25,48]:

(53) $$\left\{ \begin{array}{l} z=\pm \left(\frac{a}{2}-{\bar{h}}_{s}\right),u_{R}=0\\ z=0,\frac{\partial u_{R}}{\partial z}=0 \end{array}\right.$$

where ${\bar{h}}_{s}$ is the effective average height of the roughness element on the fracture surface, $u_{R}$ is the flow velocity of the fluid in the fracture microchannel, and the solution is:

(54) $$u_{R}=\frac{1}{2\mu }\frac{dp}{dx}\left[{\left(\frac{a}{2}-{\bar{h}}_{s}\right)}^{2}-z^{2}\right]$$

where dp/dx is the absolute value of the pressure gradient along the flow direction. Equation (54) shows that the flow velocity in the fracture network is a function of the absolute value of the pressure gradient dp/dx, the effective average height of the rough surface ${\bar{h}}_{s}$, and the distance |z| of the fluid from the center of the pipe wall. At the same time, it can be found that the closer the distance |z| of the fluid to the center of the pipe, the greater the flow velocity of the fluid, and the greater the effective average height of the roughness element, the smaller the flow velocity of the fluid. This is consistent with objective facts. The average velocity of the fluid through the rough fracture microchannel is:

(55) $${\bar{u}}_{R}=\frac{1}{b/2}\int_{0}^{\frac{b}{2}}u_{R}dz=\frac{1}{12\mu }\frac{dp}{dx}{\left(a-2{\bar{h}}_{s}\right)}^{2}$$

If ${\bar{h}}_{s}=0$, then Equation (55) is simplified to:

(56) $${\bar{u}}_{R}=\frac{1}{12\mu }\frac{dp}{dx}a^{2}$$

This is the average velocity of the fluid in the smooth fracture network. According to Equation (56), through the horizontal rough single fracture, the volume of fracture trace length l is:

(57) $$q_{R}=\int_{-\left(\frac{a}{2}-{\bar{h}}_{s}\right)}^{\frac{a}{2}-{\bar{h}}_{s}}u_{R}ldz=\frac{{\left(a-2{\bar{h}}_{s}\right)}^{3}l}{12\mu }\frac{dp}{dx}$$

Secondly, considering the spatial orientation of the fracture [19,47], the flow rate in the rough single fracture in the representative elementary volume of the fracture network is:

(58) $$q_{R}=\frac{{\left(a-2{\bar{h}}_{s}\right)}^{3}l}{12\mu }\frac{\Delta p}{L_{0}}\left(1-\cos^{2}\alpha \sin^{2}\theta \right)$$

Equation (58) shows that the volume flow rate in a single rough fracture is the function of effective opening $\;{\bar{h}}_{s}$ of the fracture surface rough element, pressure difference △p, average inclination angle θ, azimuth angle α, and fracture opening a, in the representative elementary volume of the fracture network. At the same time, it can be found that when other quantities are constant, the greater the effective opening of the rough element, the smaller the volume flow in the microchannel of a single rough fracture. In addition, when the volume flow in a single rough fracture is constant, the greater the effective opening of the rough element, the greater the pressure difference between the two ends of the fracture, and the greater the flow resistance of the fluid. According to Equation (12), the total flow of fluids through the rough fractures network representative elementary volume as:

(59) $$\begin{array}{ll} Q_{f}\left(l\right) & =\int_{l_{\min}}^{l_{\max}}\frac{{\left(a-2{\bar{h}}_{s}\right)}^{3}l}{12\mu }\frac{\Delta p}{L_{0}}\left(1-\cos^{2}\alpha \sin^{2}\theta \right)dl\\ & =\int_{l_{\min}}^{l_{\max}}\frac{a^{3}{\left(1-\varepsilon_{r}\right)}^{3}l}{12\mu }\frac{\Delta p}{L_{0}}\left(1-\cos^{2}\alpha \sin^{2}\theta \right)dl\\ & =\frac{\beta^{3}}{12\mu }\frac{D_{f}\left(1-\cos^{2}\alpha \sin^{2}\theta \right)}{4-D_{f}}\frac{\Delta p}{L_{0}}l_{\max}^{4}{\left(1-\varepsilon_{r}\right)}^{3} \end{array}$$

where $\varepsilon_{r}$ is determined by Equation (47). Equation (59) shows that the total flow in the fracture network is the function of average inclination angle θ and azimuth angle α, pressure difference △p, the relative roughness $\varepsilon_{r}$ of fracture, the fracture length fractal dimension $D_{f}$, and the maximum length $l_{\max}$ of the fracture. When other quantities are constant, the larger the relative roughness of the fracture, the smaller the flow of the fracture network, which is in line with objective physical facts.

According to Darcy’s Law:

(60) $$Q=\frac{KA_{f}}{\mu }\frac{\Delta p}{L_{0}}$$

The permeability of the fracture network is:

(61) $$K_{f}=\frac{\beta^{3}}{12A_{f}}\frac{D_{f}\left(1-\cos^{2}\alpha \sin^{2}\theta \right)}{4-D_{f}}l_{\max}^{4}{\left(1-\varepsilon_{r}\right)}^{3}$$

Substituting the $A_{f}$ value in Equation (17) into Equation (61), the permeability of the fracture network can be simplified as:

(62) $$K_{f}=\frac{\beta^{2}l_{\max}^{2}}{12}\frac{\left(2-D_{f}\right)\left(1-\cos^{2}\alpha \sin^{2}\theta \right)}{4-D_{f}}\frac{\phi_{f}}{1-\phi_{f}}{\left(1-\varepsilon_{r}\right)}^{3}$$

By substituting Equation (21) into Equation (61), the permeability of the fracture network can be expressed as:

(63) $$K_{f}=\frac{\beta^{3}D}{12}\frac{1-D_{f}}{4-D_{f}}\frac{l_{\max}^{3}\left(1-\cos^{2}\alpha \sin^{2}\theta \right)}{\left(1-\phi_{f}^{\frac{1-D_{f}}{2-D_{f}}}\right)}{\left(1-\varepsilon_{r}\right)}^{3}$$

Equation (63) shows that the permeability of the rough fracture network is a function of the structural parameters of the fracture medium (fracture length fractal dimension $D_{f}$, fracture area density D, porosity $\phi_{f}$, relative roughness $\varepsilon_{r}$, maximum fracture trace length $l_{\max}$, average fracture inclination angle θ and azimuth angle α, proportional coefficient β), and is negatively correlated with the relative roughness $\varepsilon_{r}$ of the fracture network. When $\varepsilon_{r}=0$, Equation (63) can be simplified as:

(64) $$K_{f}=\frac{\beta^{3}D}{12}\frac{1-D_{f}}{4-D_{f}}\frac{l_{\max}^{3}\left(1-\cos^{2}\alpha \sin^{2}\theta \right)}{\left(1-\phi_{f}^{\frac{1-D_{f}}{2-D_{f}}}\right)}$$

This is the permeability when the microchannel wall of the fracture network is smooth.

### 4.2. Seepage Characteristics of Pores in Rough Matrix

**Figure 6:**
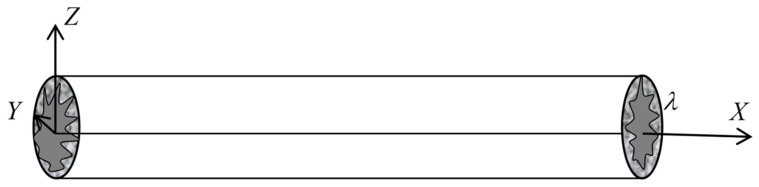
Spatial coordinates of rough straight capillaries.

Firstly, the seepage characteristics of the fluid in the rough straight capillary are studied. Assuming that the fluid flows in the X direction, only the velocity component in the X direction is not zero, and the pressure gradient along the X direction is constant, and the spatial rectangular coordinate system shown in Figure 6 is established [25]. The diameter of the capillary is λ and the radius is $r_{0}$.

Analogous to the calculation of fluid flow rate in rough fracture, the equation of motion of fluids in rough straight capillaries in fracture microchannels is [25]:

(65) $$\frac{\partial^{2}u}{\partial z^{2}}+\frac{\partial^{2}u}{\partial y^{2}}=\frac{1}{\mu }\frac{dp}{dx}$$

The no-slip boundary condition for flow is [25]:

(66) $$\left\{ \begin{array}{l} r=\pm \left(r_{0}-{\bar{h}}_{m}\right),u_{m}=0\\ r=0,\frac{\partial u_{m}}{\partial r}=0 \end{array}\right.$$

where ${\bar{h}}_{m}$ is the effective average height of roughness element on matrix pore surface, and $u_{m}$ is the flow rate of the fluid in pore microchannel. The flow rate of fluids in the rough capillary microchannel is:


(67) $$u_{m}=\frac{\left[{\left(r_{0}-{\bar{h}}_{c}\right)}^{2}-r^{2}\right]}{4\mu }\frac{dp}{dx}$$

The average flow rate is:

(68) $${\bar{u}}_{m}=\frac{1}{r_{0}}\int_{0}^{r_{0}}u_{m}dr=\frac{{\left(r_{0}-{\bar{h}}_{c}\right)}^{4}}{8\mu }\frac{dp}{dx}$$

The flow rate through a single rough straight capillary in a matrix representative elementary volume is:

(69) $$q\left(\lambda \right)=\frac{\pi }{128\mu }\frac{\Delta p}{L_{t}}{\left(\lambda -2{\bar{h}}_{ceff}\right)}^{4}=\frac{\pi }{128\mu }\frac{\Delta p}{L_{t}}\lambda^{4}{\left(1-\varepsilon_{c}\right)}^{4}$$

Second, considering the tortuosity characteristics of rough capillary, according to Equation (23) and Equation (32), the total flow of fluids through the matrix representative elementary volume is:

(70) $$Q_{m}\left(\lambda \right)=\frac{\pi L_{0}^{1-D_{T}}}{128\mu }\frac{\Delta p}{L_{0}}\frac{D_{p}}{3+D_{T}-D_{p}}\lambda_{\max}^{3+D_{T}}{\left(1-\varepsilon_{c}\right)}^{4}$$

Equation (70) is the function of fractal dimension of tortuosity, the fractal dimension of pore diameter, pressure difference, the maximum diameter of the pore, and the relative roughness of matrix pores. According to the definition of Darcy’s law and Equation (70), the permeability of rough matrices pores can be expressed as:

(71) $$K_{m}=\frac{\pi L_{0}^{1-D_{T}}}{128A_{m}}\frac{D_{p}}{3+D_{T}-D_{p}}\lambda_{\max}^{3+D_{T}}{\left(1-\varepsilon_{c}\right)}^{4}$$

Equation (71) shows that the permeability of rough matrix pores is the function of structure parameters of matrix pore (the maximum diameter $\lambda_{\max}$ of the pore, fractal dimension $D_{p}$ of the pore diameter, fractal dimension $D_{T}$ of the tortuosity, the relative roughness $\varepsilon_{c}$ of the pore). When other quantities are constant, the permeability of the rough matrix decreases with the increase in the relative roughness of the pores. When $\varepsilon_{c}=0$, Equation (71) can be simplified to:

(72) $$K_{m}=\frac{\pi L_{0}^{1-D_{T}}}{128A_{m}}\frac{D_{p}}{3+D_{T}-D_{p}}\lambda_{\max}^{3+D_{T}}$$

This is the permeability when the pore wall of the matrix is smooth.

### 4.3. Permeability Model of Rough Fractured Double Porous Media

Because the characterization unit of the fracture network is larger than that of matrix pore and contains multiple matrix units [15,19], the unit body of the dual porous media is selected as the unit body of the fracture network, there holds:

(73) $$A_{f}=n\cdot A_{m}$$

That is:

(74) $$\frac{\beta D_{f}l_{\max}^{2}}{2-D_{f}}\frac{\left(1-\phi_{f}\right)}{\phi_{f}}=n\cdot \frac{\pi D_{p}\lambda_{\max}^{2}}{4\left(2-D_{p}\right)}\frac{1-\phi_{m}}{\phi_{m}}$$

Since the flow into the porous media is equal to the flow out of the porous media [49], the total flow of fluids in the fractured dual porous media is equal to the sum of the flow in the matrix and the fracture network:

(75) $$\begin{array}{l} Q=Q_{n,m}\left(\lambda \right)+Q_{f}\left(l\right)=n\cdot Q_{m}\left(\lambda \right)+Q_{f}\left(l\right)\\ \;\;\;=n\cdot \frac{\pi L_{0}^{1-D_{T}}}{128\mu }\frac{\Delta p}{L_{0}}\frac{D_{p}}{3+D_{T}-D_{p}}\lambda_{\max}^{3+D_{T}}{\left(1-\varepsilon_{c}\right)}^{4}\\ +\frac{\beta^{3}}{12\mu }\frac{D_{f}\left(1-\cos^{2}\alpha \sin^{2}\theta \right)}{4-D_{f}}\frac{\Delta p}{L_{0}}l_{\max}^{4}{\left(1-\varepsilon_{r}\right)}^{3} \end{array}$$

The permeability of Newtonian fluids in rough fractured double porous media is defined by Darcy’s law, as:

(76) $$\begin{array}{l} K=\frac{\pi L_{0}^{1-D_{T}}}{128A_{m}}\frac{D_{p}}{3+D_{T}-D_{p}}\lambda_{\max}^{3+D_{T}}{\left(1-\varepsilon_{c}\right)}^{4}\\ +\frac{\beta^{3}}{12A_{f}}\frac{D_{f}\left(1-\cos^{2}\alpha \sin^{2}\theta \right)}{4-D_{f}}l_{\max}^{4}{\left(1-\varepsilon_{r}\right)}^{3} \end{array}$$

Equation (76) is a function of fracture network structure parameters (cross-sectional area $A_{f}$ of the fracture network characteristic unit body, fracture average inclination θ and azimuth α, fractal dimension $D_{f}$ of fracture length, relative roughness $\varepsilon_{r}$ of fracture, and proportional coefficient β) and matrix pore structure parameters (fractal dimension $D_{p}$ of pore diameter, tortuosity fractal dimension $D_{T}$, maximum diameter $\lambda_{\max}$ of pore, cross-sectional area $A_{m}$ of matrix pore characteristic unit body, and relative roughness $\varepsilon_{c}$ of pore). Meanwhile, the increase in the relative roughness of the fracture network and the relative roughness of the matrix pores lead to the decrease in permeability of fractured dual porous media.

To study the contribution of porous matrix and the fracture network to fluid permeability, dimensionless permeability $K^{+}$ is defined, namely [19,20]:

(77) $$K^{+}=K_{m}/K_{f}$$

where $K_{m}$ and $K_{f}$ represent the permeability of matrix pores and fracture networks, respectively.

## 5. Results and Discussion

**Figure 7:**
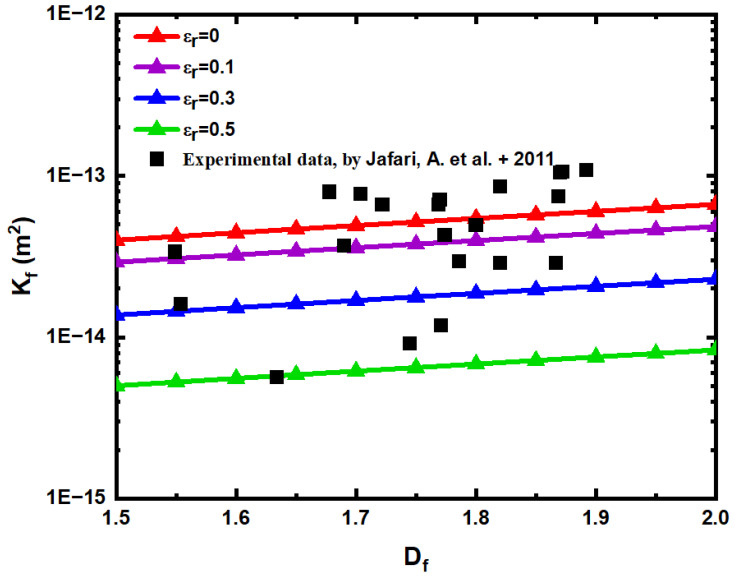
Comparison of permeability model prediction of rough fracture network and numerical simulation results [50].

Firstly, the reliability of the rough fracture network model is verified. Figure 7 compares the predicted value of the permeability model of the rough fracture network with the numerical simulation value in the literature [50] under different relative roughness. The authors of [50] selected 22 fracture types with different scales in southwestern Turkey, and the digital fracture model was imported into commercial modeling software, and the equivalent fracture network permeability was calculated by constructing a 3 D model with a network size of 100 m ×100 m ×10 m. Among them, the largest fracture length is 1 m, and the fracture inclination angle θ=0. In the calculation, take β=0.002, and calculate the predicted value of the model according to Equation (61).

According to Figure 7, the permeability of the rough fracture network increases with the increase in fractal dimension $\;D_{f}$ of fracture trace length, because the increase in fractal dimension of fracture trace length leads to the increase in fracture cross-sectional area. In addition, when the relative roughness εr of the fracture network is 0 and 0.1, the predicted permeability of the fracture network calculated by Equation (61) is 78.23% and 29.93% higher than that of the experimental simulation value. When the relative roughness εr of the fracture network is 0.3 and 0.5, respectively, the predicted permeability of the fracture network calculated by Equation (61) is 38.87% and 77.72% lower than the experimental simulation value. This shows that under the same fracture fractal dimension $\;D_{f}$, the larger the relative roughness $\varepsilon_{r}$, the smaller the fracture permeability, this is because the larger the roughness, the smaller the volume of the fracture pores, the space for fluid to flow is reduced, and the flow resistance is increased. Through the comparison of Figure 7, it is found that the established rough fracture network model is effective, and the influence of roughness on permeability is also very large.

**Figure 8:**
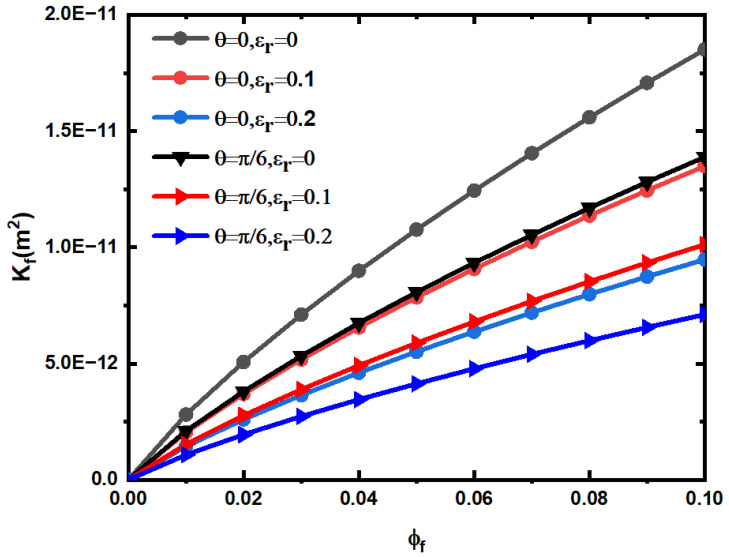
Permeability of the rough fracture network varies with fracture porosity under different inclination and relative roughness.

The influence of fracture geometry parameters on the permeability of rough fracture model is studied. Figure 8 shows the relationship between fracture network permeability and porosity under different roughness and fracture network inclination. In the calculation, β=0.01  , fracture maximum length $l_{\max}=10\;\mathrm{mm}$, and fracture spatial azimuthal angle α=0, according to Equations (13) and (62), the corresponding rough fracture network permeability is calculated. According to Figure 8, the permeability of the rough fracture network increases with the increase in fracture porosity, and the greater the inclination and relative roughness of the fracture, the smaller the permeability of the rough fracture. This is due to the increase in fracture porosity, which will increase the volume of fracture pores and increase the flowable volume of fluid, so that the fluid flow resistance decreases. The increase in inclination angle will increase the flow resistance and the increase in fracture relative roughness will reduce the effective opening of the fracture.

**Figure 9:**
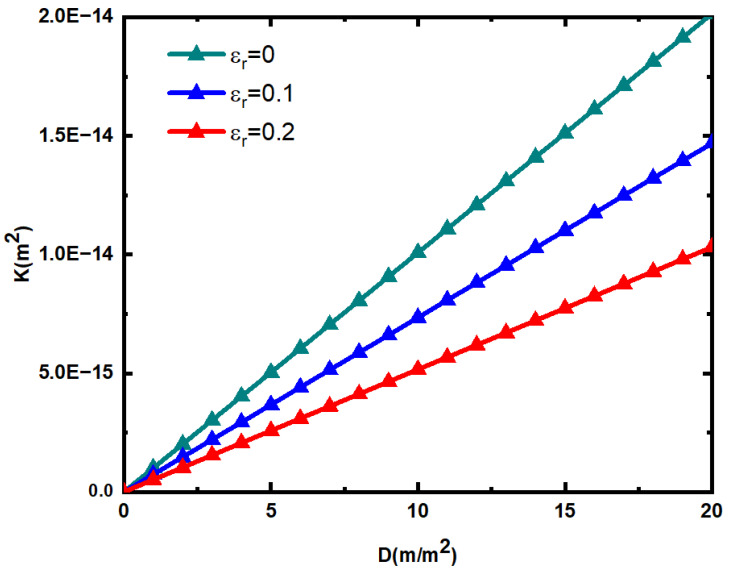
Relationship between the permeability of rough fracture network and fracture surface density and relative roughness.

Figure 9 shows the relationship between fracture network permeability and fracture surface density under different relative roughness, in the calculation, the maximum fracture length $l_{\max}=10\;\mathrm{mm}\;\;$, the fracture dip angle θ=45°, the average porosity of the fracture network is 0.018, the average fractal dimension of the fracture network is 1.3, and β=0.01. According to Equation (63), the corresponding rough fracture network permeability is calculated. According to Figure 9, the permeability of the rough fracture network increases with the increase in the fracture surface density. The greater the relative roughness of the rough fracture network, the smaller the permeability, and the effect of relative roughness on the fracture permeability is very obvious. This is due to the increase in fracture surface density will increase the length of fracture trace and reduce the fluid flow resistance. The increase in the relative roughness of the fracture will increase the flow resistance of the fluid.

**Figure 10:**
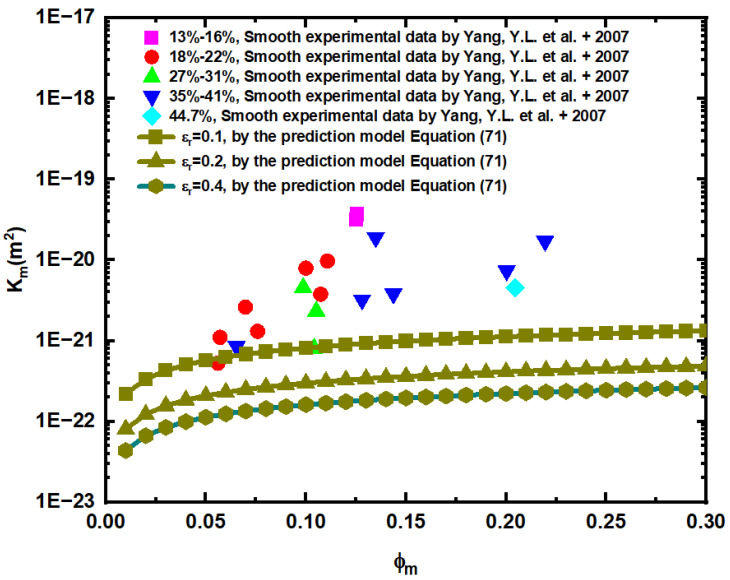
Comparison of predicted pore permeability of rough matrix with experimental data [51]. The samples are classified by clay content.

Figure 10 is a comparison of the predicted pore permeability of the rough matrix with the numerical simulation values of smooth matrix permeability in [51]. The authors of [51] measured the permeability and other data of 30 natural mudstones collected from oil wells at depths of two to three km, among them, the permeability was measured on disks with a thickness of 0.005–0.008 m and a diameter of 0.0254 m. The porosity is between 0.06 and 0.27, the average radius of the pore is between 2.8 and 1403.4 nm, and no fractures are found. So the maximum pore diameter is 2806.8 nm and the minimum is 5.6 nm. Equation (26) can be used to calculate $D_{p}$, Equations (31), (34) and (35) can be used to calculate $D_{T}$, and Equation (71) can be used to calculate the corresponding matrix permeability prediction value.

Figure 10 shows that when the relative roughness of matrix pores is 0.1, 0.2, and 0.4, the predicted values of matrix pore permeability calculated by Equation (71) are 66.4%, 79.05%, and 93.37% lower than the experimental simulation data, respectively. Through comparison, it is found that the predicted value of the rough pore permeability model is smaller than the experimental data, and the greater the relative roughness of the pore, the smaller the pore permeability, which further illustrates the effectiveness of this model.

**Figure 11:**
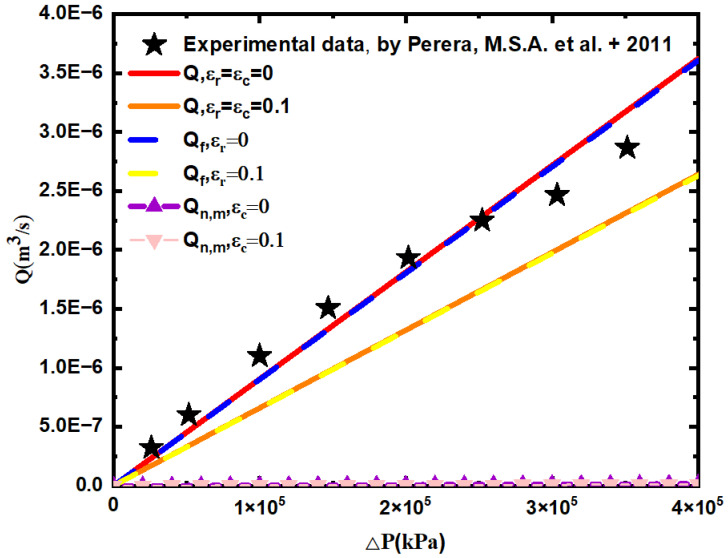
Comparison of predicted and experimental data [52] of Newtonian fluid flow at 25 °C temperature and 500 kPa confining pressure.

Figure 11 shows the relationship between the total flow and the pressure difference for a Newtonian fluid, when the temperature is 25 °C and the confining pressure is 500 Kpa, the total flow prediction of a Newtonian fluid through the fractured double porous media, and the flow prediction through the fracture network and the matrix pore are compared with the experimental data [52], which simulated the smooth dual porous media. In the calculation, the average inclination angle of the fracture is θ=0°, the minimum diameter of the matrix capillary is 2 nm, the viscosity coefficient of the water is $\mu =1.12\times 10^{-3}\;\mathrm{Pas}$, and the maximum length of the fracture is 10 mm. The relationship between the maximum and minimum pore diameter and the fracture length is $\lambda_{\min}/\lambda_{\max}=0.001$, and $l_{\min}/l_{\max}=0.001$. According to the literature [52], the matrix porosity is $\phi_{m}=0.25$, and the fracture porosity is  1/100 of the matrix, that is $\phi_{f}=0.0025$. Then, the corresponding flow rates of the fracture network, matrix pores, and fractured dual porous media are calculated according to Equations (59), (70), and (75).

According to Figure 11, when the relative roughness of the matrix pore and the fracture network are $\varepsilon_{r}=\varepsilon_{c}=0$ and ε=rεc=0.1, the total flow prediction value of the fractured dual porous media calculated by Equation (75) is 7.53% and 32.62% lower than the experimental simulation values, respectively, This indicates that the flow rate of rough fractal model is lower than the total flow of smooth fractal model and the experimental data, and further shows that the roughness of the porous medium has a greater influence on fluid flow. This is because when the relative roughness increases, the flow resistance of the fluid increases, resulting in a decrease in the total flow through it, this is the same as the actual physical situation and further indicates the validity of the established model. At the same time, when the relative roughness of the fracture network is 0, 0.1, the predicted value of the total fracture network flow calculated by Equation (70) is 7.94% and 32.98% lower than the experimental simulation value, respectively, and it can be found that the flow of the fracture network is almost the same as that of the fractured dual porous media, and the flow of the matrix pore is much smaller than the flow of the fracture network, and the relative roughness of the matrix pore has little effect on the matrix flow.

The influence of structural parameters on permeability of fractured dual porous media is studied below. When the structural parameters ($\phi_{f}$, $\phi_{m}$, $D_{p}$, $D_{T}$, β, α, θ, $L_{0}$) of the fractured dual porous media are known, the permeability prediction can be calculated using Equation (76). The structural parameters in the calculation process take the values in the literature [19], where the maximum diameter of the matrix pores is λ max=2 μm, the average inclination angle of the fracture network is θ =π/6, the maximum value of the fracture length is $l_{\max}\;=0.010\;m$, the average azimuth angle of the fracture network is α=0, the linear length of the matrix pore is $L_{0}=0.1\;m$, and the ratio of the minimum and the maximum pore diameters $\lambda_{\min}/\lambda_{\max}$ and the ratio of the minimum and maximum fracture length $l_{\min}/\;l_{\max}$ are all 0.001.

Firstly, when the structural parameters of matrix porous medium are determined, the influence of fracture network structural parameters on the permeability of fractured dual porous media is studied.

**Figure 12:**
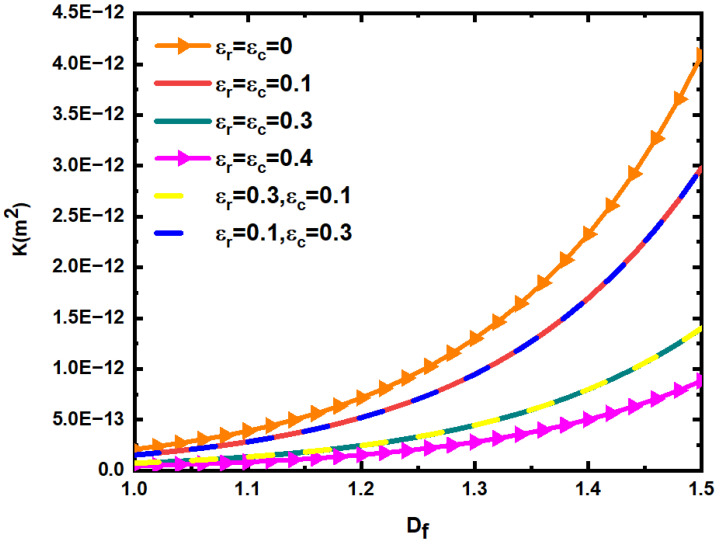
Relationship between permeability K of fractured dual porous media and fracture length fractal dimension *D_f_* with different relative roughness.

Figure 12 shows the change of permeability K of fractured dual porous media when the relative roughness $\varepsilon_{r}$ and $\varepsilon_{c}$ of the fracture network and matrix pores and the fractal dimension $D_{f}$ of fracture length take different values. In the calculation, take β=0.01, $\phi_{m}=0.25$. According to Equations (14), (26) and (35), the fractal dimension $D_{p}$ of the pore diameter, the porosity $\phi_{f}$ of the fracture network, and the fractal dimension $D_{T}$ of the tortuosity can be determined, and the cross-sectional area $A_{f}$, $A_{m}$ of the fracture and matrix characterization element can be determined by Equations (17) and (30). Then, the corresponding permeability of fractured dual porous media can be determined according to Equation (76). According to Figure 12, the permeability of fractured dual porous media increases with the increase in fractal dimension of fracture trace length. When the fractal dimension of fracture trace length is constant, the permeability of dual porous media decreases with the increase in the relative roughness of fracture and matrix. At the same time, it can also be found that when the values of $\varepsilon_{r},\;\;\varepsilon_{c}$ are 0.3, 0.1 and 0.3, 0.3, respectively, the permeability of the fractured dual porous media is almost equal, indicating that the relative roughness of the matrix pores has little effect on the seepage of the fluid. When $\varepsilon_{r},\;\;\varepsilon_{c}$ are 0.1 and 0.3, respectively, the permeability of fractured dual porous media is far greater than that of $\varepsilon_{r},\;\;\varepsilon_{c}$ is 0.3 and 0.3, respectively, and is almost equal to that of $\varepsilon_{r},\;\;\varepsilon_{c}$ is 0.1 and 0.1, respectively. This indicates that the relative roughness of the fracture network has a very large influence on the seepage of the fluid, which further indicates that the main function of matrix pores is storage, and the fracture network can be the main channel of fluid seepage. Thus, when studying the permeability of rough fractured dual porous media, the influence of matrix roughness can be ignored, considering that the matrix pores are smooth.

**Figure 13:**
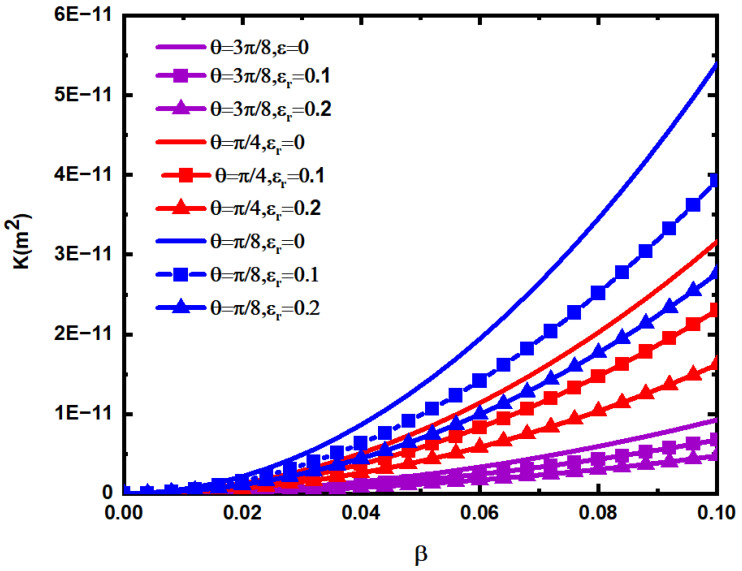
Relationship between the permeability K of the fractured dual porous media and the ratio β of fracture opening to trace length, the relative roughness $\varepsilon_{r}$ of the fracture network, and the average inclination angle θ.

Figure 13 shows the variation law of the permeability of the fractured dual porous media when the ratio β of fracture opening to trace length, the relative roughness $\varepsilon_{r}$ of the fracture network, and the average inclination θ change. In the calculation, take $\phi_{m}=0.25$, $\;\phi_{f}=0.0025$. According to Figure 13, the permeability K of fractured dual porous media increases with the increase in the ratio β of fracture opening to the fracture length, and the greater the β, the faster the increase in K . At the same time, the greater the average inclination angle θ and relative roughness $\varepsilon_{r}$ of the fracture network, the smaller the permeability K  of the fractured dual porous media, and the slower its increase rate with the increase in β. This indicates that the greater the average inclination angle and the relative roughness of the fracture network, the greater the flow resistance of the fluid.

Secondly, when the structural parameters of the fracture parameters are determined, the influence of matrix pore structural parameters on fluid permeability is studied. According to Equations (76) and (77), when the structural parameters of the fracture network are determined, the dimensionless permeability $K^{+}$ is only affected by matrix pore structural parameters.

**Figure 14:**
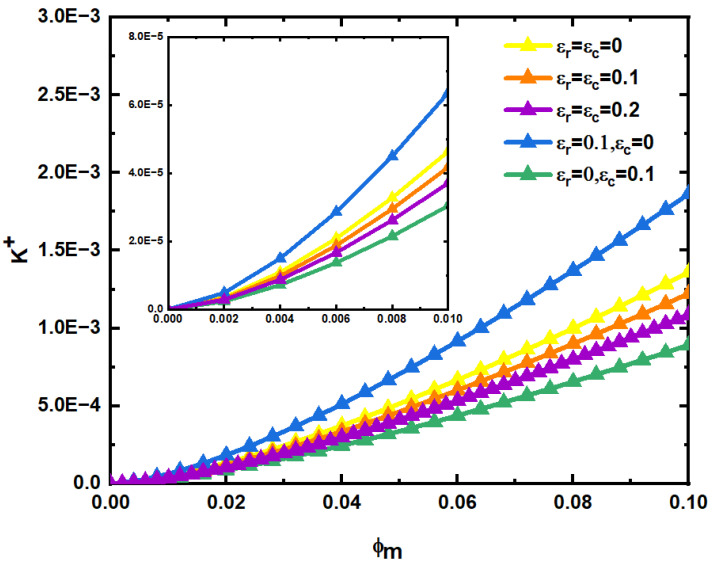
Relationship between dimensionless permeability $K^{+}$ and matrix porosity $\phi_{m}$, fracture network and matrix relative roughness $\varepsilon_{r}$, $\varepsilon_{c}$.

Figure 14 shows the changing trend of dimensionless permeability $K^{+}$ with matrix porosity $\phi_{m}$, fracture network and matrix relative roughness $\varepsilon_{r}$, $\varepsilon_{c}$. In the calculation, the structural parameters of the fracture network are taken from the values in [19]. As can be seen from Figure 14, when the relative roughness $\varepsilon_{r}$, $\varepsilon_{c}$ of the fracture network and the matrix are constant, $K^{+}$ increases with the increase in the matrix porosity $\phi_{m}$, and when relative roughness $\varepsilon_{r}$, $\varepsilon_{c}$ increases at the same time, the dimensionless permeability $K^{+}$ decreases, indicating that the decreases rate of matrix permeability is less than that of fracture network permeability. This further shows that relative roughness has a greater impact on fracture networks. In addition, the permeability of matrix pores is much smaller than that of the fracture network, no matter how the relative roughness changes, in other words, the main function of the matrix pores is storage, and fracture networks serve as the main channels for fluid flow. Additionally, because the fluid has a dissolution effect, the pores of the porous medium will gradually become large caves, and the fractures will also become large fractures.

## 6. Conclusions

Based on the hypothesis that the fracture network is composed of rectangular pipes of different sizes and satisfies the fractal distribution, the matrix pores are composed of curved capillaries with circular cross-sections, and the roughness is characterized by small cones. The fractal theory is used to deduce the total flow rate and permeability model of a Newtonian fluid in rough fractured dual porous media, and the relationship between the permeability of dual porous media and the fractal dimension $\left(D_{f},D_{p},D_{T}\right)$, relative roughness $\;\left(\varepsilon_{r},\varepsilon_{c}\right)$, fracture network inclination θ, fracture porosity $\phi_{f}$, matrix porosity $\phi_{m}$ and other parameters of the fracture network and matrix pores is obtained. By comparing with the experimental data, it is found that the total flow rate of rough fractured dual porous media decreases with the increase in relative roughness and is lower than the experimental data, indicating the reliability of the established model. Through comparison, it is found that the permeability of the fractured dual porous media increases with the increase in the fracture porosity, the ratio of fracture opening to trace length, and the porosity of the matrix; and the greater the relative roughness of the fracture and matrix and fracture inclination angle, the smaller the permeability of the fractured dual porous media. In addition, the relative roughness of the fracture network has a much greater influence on the permeability of fractured dual porous media than the relative roughness of matrix pores; therefore, the roughness of the matrix pores can be ignored in this study, assuming that it is smooth.

## Data Availability

This article details the data and results covered by this study.

## Nomenclature

a	The opening of the fracture
l	The trace length of the fracture
θ	The average inclination angle of fractures
α	The average azimuth angle of fractures
β	The ratio of fracture opening to fracture trace length
λ	The diameter of the capillary
$D_{f}$	The fractal dimension of fracture trace length
$D_{p}$	The fractal dimension of pore diameter
$\phi_{s}$	The percentage of the total bottom area of all cones to the total area of fracture surface in the fracture network representative elementary volume
$\phi_{f}$	The porosity of the fracture network
$\phi_{m}$	The porosity of the matrix pores
${\bar{h}}_{s}$	The effective average height of rough elements in fracture
${\bar{h}}_{c}$	The effective average height of rough elements on rough capillaries
$\varepsilon_{r}$	The relative roughness of the fracture surface
$\varepsilon_{c}$	The relative roughness of the rough capillary bundle
$\bar{\tau }$	The average tortuosity of the capillary
$\bar{\lambda }$	The average diameter of the pores
$Q_{f}$	The total flow of fluids through the rough fractures network representative elementary volume
$Q_{m}$	The total flow of fluids through the matrix representative elementary volume
$K_{f}$	The permeability of the fracture network
$K_{m}$	The permeability of rough matrices pores
$L_{0}$	The characteristic length of the capillary
$L_{t}$	The actual length of the capillary
d	The bottom diameter of the cone
h	The height of the cone
σ	The ratio of the height of the cone to the diameter of the base
$D_{T}$	The tortuosity fractal dimension
$D_{s}$	The fractal dimension of the bottom diameter of the cone
$S_{1}$	The total bottom area of all cones in representative elementary volume
$S_{0}$	The total area of fracture surface in representative elementary volume
$A_{pf}$	The total area of fracture pores
$A_{p}$	The total matrix pores area
$A_{f}$	The cross-section area of the representative elementary volume of the fracture network
$A_{m}$	The cross-sectional area of matrix representative elementary volume
$S_{i}$	The bottom area of a small cone
$V_{t}$	The total volume of all cones in the representative elementary volume
$V_{i}$	The volume of a small cone
D	The fracture areal density
Q	The total flow of fluids in the fractured dual porous media
K	The permeability of a Newtonian fluid in rough fractured double porous media
$K^{+}$	The dimensionless permeability
